# Small Dense LDL Level and LDL/HDL Distribution in Acute Coronary Syndrome Patients

**DOI:** 10.3390/biomedicines11041198

**Published:** 2023-04-18

**Authors:** Alyann Otrante, Abdelghani Bounafaa, Hicham Berrougui, Abdel-Khalid Essamadi, Michel Nguyen, Tamàs Fülöp, Abdelouahed Khalil

**Affiliations:** 1 Geriatrics Unit, Department of Medicine, Faculty of Medicine and Biological Sciences, University of Sherbrooke, Sherbrooke, QC J1K 2R1, Canada; 2 Laboratory of Biochemistry, Neuroscience, Natural Resources and Environment, Faculty of Sciences and Technology, Hassan First University of Settat, Settat 26002, Morocco; 3 Department of Biology, Polydisciplinary Faculty, Sultan Moulay Sliman University, Beni-Mellal 23000, Morocco; 4 Cardiology Unit, Department of Medicine, Faculty of Medicine and Biological Sciences, University of Sherbrooke, Sherbrooke, QC J1H 4N4, Canada

**Keywords:** acute coronary syndrome, sdLDL, ROC curve, LDL and HDL subclass distribution

## Abstract

This study aimed to determine the size and distribution of LDL and HDL particles in North African acute coronary syndrome (ACS) patients and to compare the level of small dense LDL (sdLDL) to other markers used in cardiovascular risk prediction. Methods: A total of 205 ACS patients and 100 healthy control subjects were enrolled. LDL particle size and LDL and HDL subclass distributions were measured using Quantimetric Lipoprint^®^ linear polyacrylamide gel electrophoresis. Lipid ratios (total cholesterol, LDL cholesterol, non-HDL cholesterol, and HDL cholesterol) were determined to calculate the atherogenic index of plasma (AIP), the atherogenic coefficient (AC), Castelli’s Risk-I (CR-I), and Castelli’s Risk-II (CR-II). Receiver operating characteristic (ROC) curve analyses and area under the curve (AUC) were used to assess the predictive value of sdLDL as a marker for cardiovascular disease. Results: The ACS patients, compared to the healthy control subjects, displayed an alteration of LDL particle distribution, with a significant increase in sdLDL serum concentrations (0.303 ± 0.478 mmol/L vs. 0.0225 ± 0.043 mmol/L, respectively, *p* < 0.001). The sdLDL levels had a high discrimination accuracy [AUC = 0.847 ± 0.0353 (95% CI 0.778 to 0.916, *p* < 0.0001)]. The best predictive cutoff value of ACS determined with the maximum Youden index (J) [(sensitivity + specificity) − 1 = 0.60] was 0.038 mmol/L. A Spearman correlation analysis showed that sdLDL levels were moderately but significantly and positively correlated with AC and CR-I (r = 0.37, *p* < 0.001) and weakly but significantly correlated with PAI and CR-II; r = 0.32 (*p* < 0.001) and r = 0.30 (*p* < 0.008), respectively. The subclass distribution of HDL particles from ACS patients was also altered, with a decrease in large HDL particles and an increase in small HDL particles compared to HDL from healthy control subjects. Conclusion: Due to their high atherogenicity, sdLDL levels could be used as a valuable marker for the prediction cardiovascular events.

## 1. Introduction

High LDL (low-density lipoproteins) cholesterol levels are associated with a high risk of coronary heart disease (CHD), whereas HDL (high-density lipoproteins) cholesterol levels are inversely associated with the risk of CHD. Current cholesterol guidelines target reduction in LDL cholesterol levels as an efficient strategy for people at higher risk of CVD. However, even at normal LDL levels, LDL particle size is emerging as a good marker for carotid atherosclerosis, particularly in the presence of cardiovascular risk factors such as dyslipidemia, diabetes, hypertension, chronic kidney disease, and smoking [1].

LDL cholesterol corresponds to a heterogeneous group of particles that vary in size, density, lipid composition, electrical charge, and functional properties. The distribution of LDL particles is affected by both genetic and environmental factors. Subjects presenting elevated small dense LDL (sdLDL) levels are at a higher risk of CHD than subjects with higher large buoyant LDL (lbLDL) levels [2,3]. This atherogenic effect of sdLDL particles has been principally attributed to their higher susceptibility to oxidation, apolipoprotein B glycation, and increased arterial wall uptake [4,5]. However, other factors that may be involved in the high atherogenicity of sdLDL remain to be clarified.

Clinical studies have shown that sdLDL levels are independently associated with the development and progression of atherosclerosis, suggesting that they could be used as a biomarker of cardiovascular risk and for the reclassification of patients from low-risk to higher-risk classes [6]. Consistent with this, different threshold levels of sdLDL have been associated with an increase in CVD risk. However, the results from the literature indicate that these sdLDL threshold values vary widely [6,7,8].

Several methods have been developed to separate LDL fractions and to quantify sdLDL levels and LDL particle sizes [9]. However, depending on the method used, different numbers of fractions and various nomenclatures have been attributed to LDL subclasses [9]. Moreover, depending on the method used, the level of sdLDL in plasma can vary from 6% to 93% [8]. This indicates a clear need to establish a standard method to better quantify sdLDL levels, which may facilitate the comparison of data from different studies and possibly identify the threshold level of sdLDL for increased cardiovascular risk. The Lipoprint^®^ Quantimetric assay is, to date, the only technique approved by the FDA (Food and Drug Administration) for clinical use in cardiovascular risk assessment through the quantification of the different LDL and HDL subclasses.

This study aimed to determine the size and distribution of LDL and HDL particles in North African acute coronary syndrome (ACS) patients and to compare the level of sdLDL to other markers used in cardiovascular risk prediction.

## 2. Materials and Methods

### 2.1. Subjects

A total of 305 subjects were enrolled and distributed into two groups according to their health status. One group consisted of 100 healthy control subjects (52 men and 48 women, mean age 55.07 ± 5.88). They were all healthy non-smokers and were not taking any medication. The second group consisted of 205 ACS patients (125 men and 80 women, mean age 57.47 ± 9.59 years). All the ACS patients met the criteria for ACS, which was characterized using ECGs to detect NSTEMI, STEMI, and unstable angina. Instrumental examination, which included coronary angiography and echocardiography, was used to detect acute myocardial infarction. Exclusion criteria included renal failure (creatinine clearance <40 mL/min), dysthyroidism, and hormonal treatments. Arterial blood pressure, lipid profiles (LDL, HDL, total cholesterol, triglycerides), C-reactive protein (CRP), and glycemia levels were determined. The physical and biochemical characteristics of all the participants (healthy control subjects and ACS patients) are listed in Table 1. The present study was conducted according to Declaration of Helsinki guidelines. All participants provided written informed consent prior to taking part in the present study. The protocol was approved by the Ethics Committee of the Sherbrooke University Hospital Center (#2019-2790).

### 2.2. Blood Collection

Blood samples (30 mL) were collected in EDTA from each participant after overnight fasting. For healthy patients, the blood samples were taken during their medical check-ups visit, whereas for ACS patients, the blood samples were collected at admission to the cardiology unit. Plasma glucose and serum lipid profiles (total cholesterol, LDL cholesterol, HDL cholesterol, and triglycerides) were measured using an automatic biochemistry analyzer. Plasma was separated using low-speed centrifugation (1500× *g*) and was stored at –80 °C until used for the measurement of LDL and HDL particle size and distribution.

### 2.3. LDL Subfraction Analysis

The LDL particle size analysis was carried out with linear 3% polyacrylamide gel tubes using the Lipoprint^®^ LDL system (Quantimetrix^®^, Redondo Beach, CA, USA) according to the manufacturer’s instructions and as previously described [10]. In brief, 25 μL of each sample was mixed with 200 μL of gel loading Lipoprint^®^ and deposited on the top of the 3% polyacrylamide gels. The gel tubes were maintained under UV light at room temperature for 30 min to induce photopolymerization. They then underwent electrophoresis for 60 min at 3 mA. A sample provided by the manufacturer was used for EDTA and plasma samples were used for quality control. Image scanning was performed using an ArtixMaker M2 digital scanner (Mikrotek, Co., Hsinchu, Taiwan), and the LDL subfractions were quantified using the Lipoware software program (Quantimetrix). Based on this analysis, very-low-density lipoprotein (VLDL) remained at the origin (retention factor; Rf = 0.0), whereas HDL migrated to the front (Rf = 1.0). The LDL were resolved into a maximum of seven LDL subfractions (LDL1 to LDL7). The same procedure, with minor modifications, was used for the HDL particle distribution analysis. The intra-assay precision for LDL and HDL quantification is, respectively, 1.20% and 1.87%, whereas the inter-assay precision is 1.26% for LDL and 3.15% for HDL. The mean intra-assay precision for the sdLDL (LDL_1_ to LDL_7_) is 2.62%

### 2.4. Atherogenic Indices

The following atherogenic indices were determined for each participant (healthy control subjects and ACS patients): the AIP, which corresponds to the logarithmically transformed ratio of molar concentrations of triglycerides (TG) to HDL cholesterol [log [TG]/[HDLc]]; the AC, which corresponds to the ratio of non-cholesterol HDL to HDL cholesterol [[total cholesterol−HDLc]/[HDLc]]; CR-I, which corresponds to the total cholesterol (TC) to HDL cholesterol ratio [TC/HDLc]; and CR-II, which corresponds to the LDL cholesterol to HDL cholesterol ratio [[LDLc]/[HDLc]]. The relevance of using these indices, both for monitoring lipid profiles and in daily practice for monitoring the risk of CVD in patients at risk, has been widely demonstrated [11,12,13].

### 2.5. Statistical Analysis

Continuous variables are expressed as means ± standard deviation (SD) and were analyzed using the Mann–Whitney U test to compare the distribution of LDL particles in healthy control subjects and ACS patients. Categorical variables were described as proportions and were analyzed using the χ2-test. Receiver operating characteristic (ROC) curve analysis was used to determine discrimination accuracy. The area under the curve (AUC) was used to calculate the predictive power of each atherogenic index (i.e., sdLDL, AIP, AC CR-I, and CR-II). DeLong’s test was performed for the statistical comparison of ROC-AUCs. The maximum Youden’s index value was used to calculate the optimal cutoff for each parameter. A Spearman correlation analysis was used to determine the degree of association between two variables. *p*-values of less than or equal to 0.05 were considered significant. All tests were two-sided. Statistical analyses were performed using GraphPad Prism software, version 8.0 (GraphPad Software Inc., La Jolla, CA, USA).

## 3. Results

Table 1 presents the clinical and biochemical parameters of the participants in both groups. The two groups were comparable with respect to the proportion of men and women (*p* < 0.14). The ACS group was slightly but significantly older than the healthy control group (mean age 57.47 ± 9.59 vs. 55.07 ± 5.88 years, respectively, *p* < 0.022). The two groups also presented significant differences with respect to their BMI. Most ACS patients were obese or overweight (mean BMI = 27.25 ± 3.73) compared to the healthy control group (mean BMI = 24.33 ± 2.34 Kg/m^2^) (*p* < 0.0001). The ACS patients also presented significantly higher systolic and diastolic blood pressures (*p* < 0.0001 and *p* < 0.0241, respectively) than the healthy control subjects, while 36% of the ACS patients were hypertensive, 40% were smokers, and 27.11% had a familial history of cardiovascular diseases and only 5% of ACS patients were under statins.

With respect to the lipid profile, the ACS patients presented significantly higher levels of total cholesterol (*p* < 0.0001), LDL cholesterol (*p* < 0.0015), and triglycerides (*p* < 0.0001) and had lower HDL cholesterol levels compared to the healthy control subjects (*p* < 0.0002). Moreover, the ACS patients had higher blood glucose levels (*p* < 0.0001). In addition, more than half of the ACS patients were diabetic.

**Figure 1:**
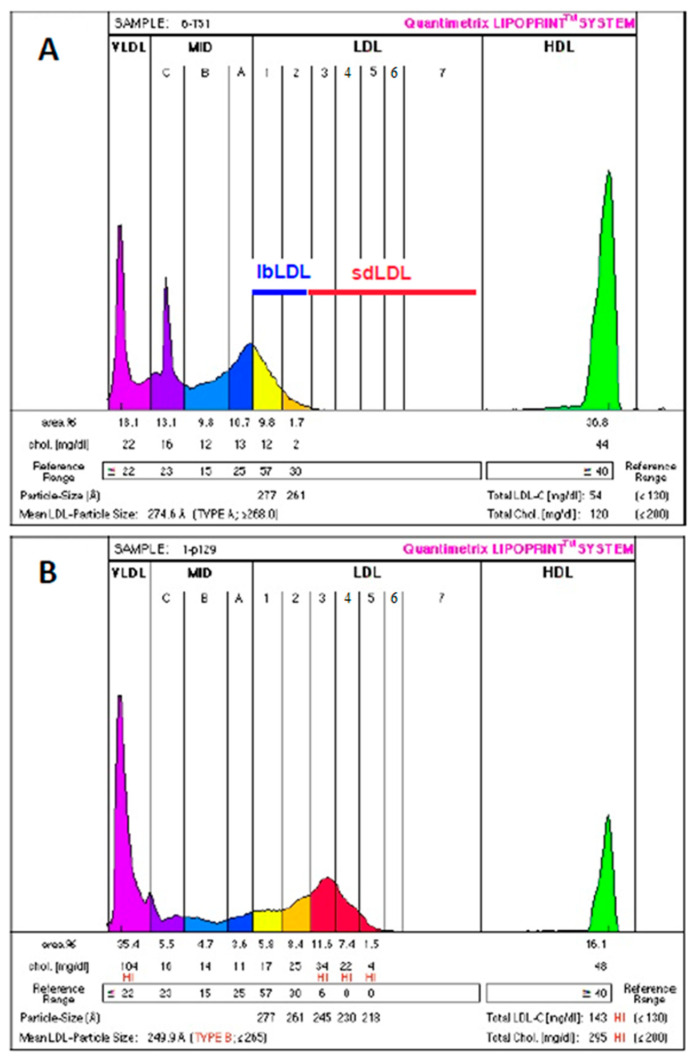
Subclass distribution profile of LDL using the Quantimetrix^®^ Lipoprint^®^ LDL System. EDTA-plasma samples were used for the LDL particles size analysis. LDL were separated into seven fractions (LDL1 to LDL7), of which fractions 1 and 2 corresponded to large buoyant LDL (lbLDL) and fractions 3 to 7 corresponded to small dense LDL (sdLDL). LDL were obtained from healthy control subjects (**A**) and from ACS patients (**B**). Subclass distribution is presented in concentration of cholesterol (mg/dL) and in % (area %). Particle size is expressed in angstroms (Å).

**Figure 2:**
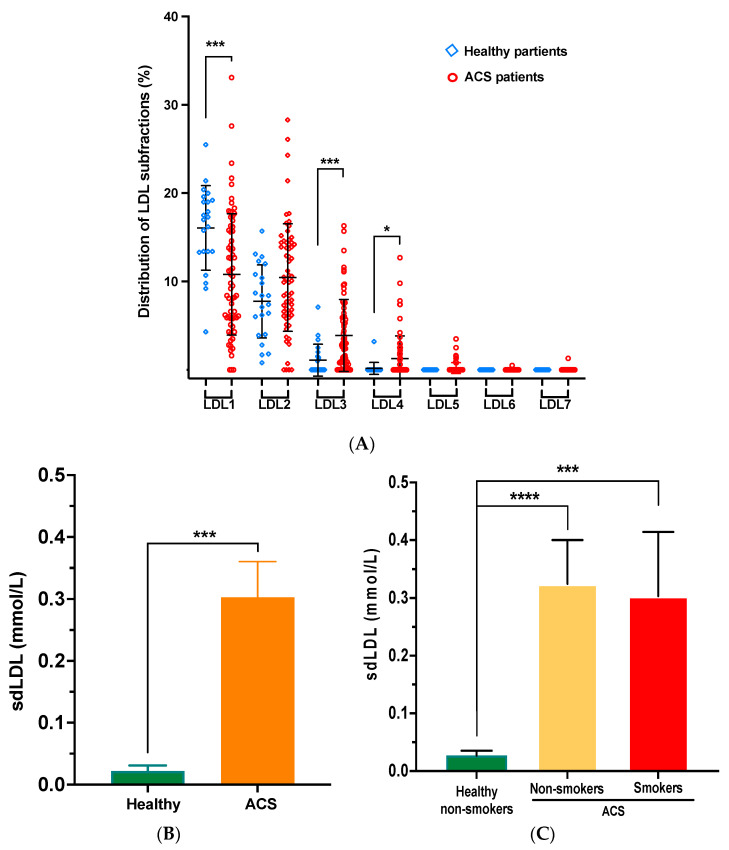
(**A**) Lipoprotein subclass distribution of LDL from ACS patients compared to healthy control subjects. (**B**) Measurement of sdLDL-forming LDL obtained from ACS patients compared to healthy control subjects. (**C**) Measurement of sdLDL-forming LDL obtained from non-smoker and smoker ACS patients compared to healthy (non-smoker) control subjects. LDL subclass distribution analysis and sdLDL quantification were determined using the Quantimetrix^®^ Lipoprint^®^ LDL System.**** *p* < 0.0001 *** *p* < 0.0003 and * *p* < 0.01.

First, we were interested in comparing the LDL distribution profiles according to the health status of the participants (ACS patients vs. healthy control subjects). Figure 1 presents, by way of example, the subclass distribution profile of LDL obtained from the healthy control subjects (Figure 1A) compared to that of LDL obtained from the ACS patients (Figure 1B). The healthy control subjects presented a distribution profile dominated by lbLDL particles corresponding to two fractions: LDL1 and LDL2. Conversely, the ACS patients presented an LDL distribution profile dominated by sdLDL particles that corresponded to five fractions: LDL3 to LDL7 (Figure 1B). Quantitatively, the ACS patients had significantly higher plasma sdLDL levels than the healthy control subjects (0.303 ± 0.478 mmol/L vs. 0.0225 ± 0.043 mmol/L, respectively) (Figure 2A,B). No significant difference was observed in the sdLDL levels between non-smoker and smoker ACS patients. However, both subgroups (non-smoker and smoker ACS patients) have significantly higher sdLDL levels compared to the control group (Figure 2C). The LDL of the ACS patients was also characterized by a lower particle size compared to the healthy control subjects (25.59 ± 0.57 nm and 27.1 ± 0.08 nm, respectively).

We also compared the two groups according to their level of cardiovascular risk. We determined the various atherogenic indices generally used for the prognosis and diagnosis of CVD (PAI, AC, CR-I, and CR-II) (Table 1). Although the four cardiovascular risk indices of the healthy control subjects were in the normal range (PAI < 0.1, AC < 3, CR-I < 5, and CR-II < 3), 77.07% of the ACS patients presented a significantly higher PAI (0.32 ± 0.28, *p* < 0.0001), 54% presented a higher AC (4.24 ± 1.94, *p* < 0.001), 87.31% presented a higher CR-I (5.24 ± 1.94, *p* < 0.001), and 65.85% presented a higher CR-II (4.06 ± 1.94, *p* < 0.001), which was consistent with their health status (Table 1).

The Spearman correlation analysis showed that the sdLDL levels were moderately but significantly and positively correlated with the AC and the CR-I indices (supplementary data Figure S1) (r = 0.37, *p* < 0.001). The plasma sdLDL levels were positively and significantly, although weakly, correlated with the PAI and CR-II results (r = 0.32 (*p* < 0.001) and r = 0.30, respectively, *p* < 0.008).

**Figure 3:**
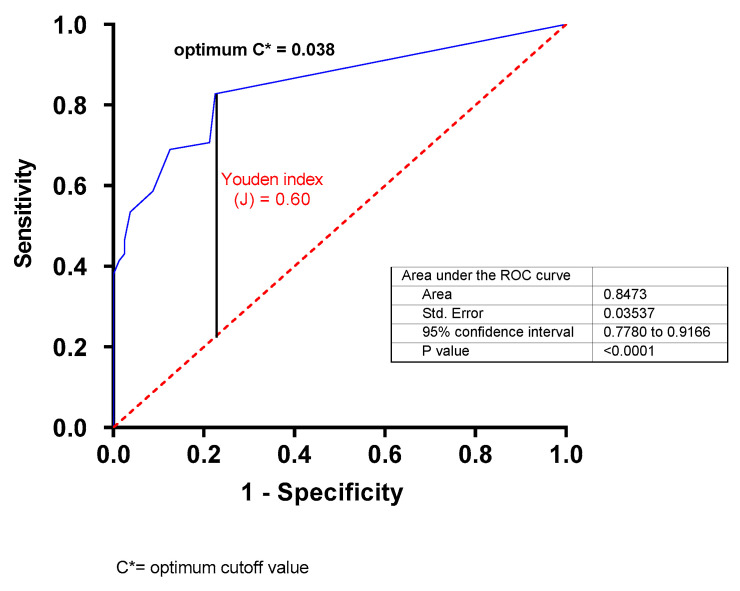
Receiver operating characteristic (ROC) curves exhibiting the discriminatory power of sdLDL. C*: optimum cutoff value of ACS determined using the maximum Youden index (J) [(sensitivity + specificity) − 1 = 0.60].

To further determine whether sdLDL levels could predict the risk of ACS, a ROC curve was plotted (Figure 3). The results showed that the discrimination accuracy of the sdLDL levels was equal to 0.8437 ± 0.0353 (95% CI 0.778 to 0.916, *p* < 0.0001) (Figure 3). The best predictive cutoff value of ACS, determined using the maximum Youden index (J) [(sensitivity + specificity) − 1 = 0.60], was 0.038 mmol/L.

**Figure 4:**
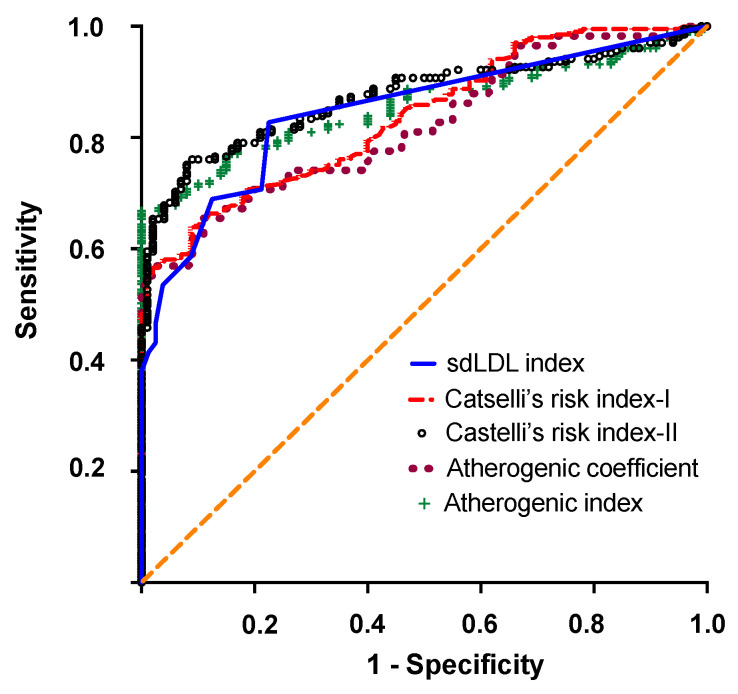
Receiver operating characteristic (ROC) curves showing the discriminatory power of sdLDL, AIP, AC, CR-I, and CRII.

The result of the ROC analysis obtained with sdLDL was compared to those of the other atherogenic indices. Figure 4 presents a merging of the ROC curves obtained for each of the four atherogenic indices. The AUC values for each marker are summarized in Table 2.

Interestingly, the high discrimination accuracy obtained with sdLDL (0.8437 95% CI 0.7780 to 0.9166) was comparable to that obtained for AI (0.8566 95% CI 0.8158 to 0.8975), AC (0.8362 9% CI 0.7932 to 0.8793), CR-I (0.8362 95% CI 0.7932 to 0.8793) and CR-II (0.8730 95% CI 0.7780 to 0.9166). DeLong’s test was performed to compare ROC curves and to test the statistical significance of the difference between the ROC-AUCs of the studied atherogenic indices [14]. No significant difference was observed between the AUC of sdLDL and AUCs of the fourth atherogenic indices (AI, AC, CR-I and CR-II). Table 3 presents the optimum cutoff points for each atherogenic biomarker at the highest Youden’s index (maximum sensitivity and specificity). The optimal cutoff points were AI = 0.135, AC = 4.350, CI-I = 3.890, CI-II = 3.195, and sdLDL = 0.038 mmol/L (Table 3).

**Figure 5:**
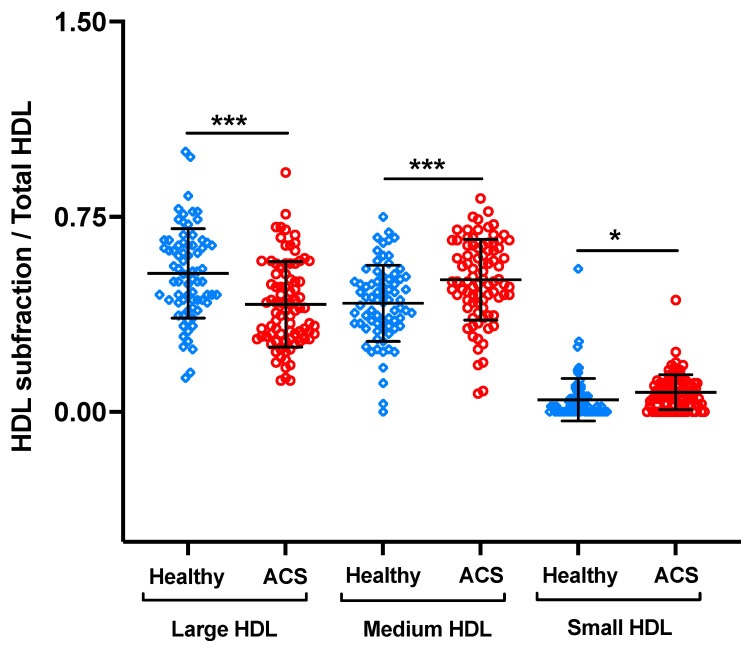
Lipoprotein subclass distribution of HDL from ACS patients compared to healthy control subjects. HDL subclass distribution analysis was determined using the Quantimetrix^®^ Lipoprint^®^ HDL System. *** *p* < 0.0003 and * *p* < 0.01.

The subclass distribution of HDLs was also analyzed. HDL obtained from ACS patients exhibited a significant decrease in large HDL levels compared to HDL from healthy control subjects (41.60 ± 16.08 vs. 53.12 ± 17.26%, respectively, *p* < 0.0001) (Figure 5). Conversely, intermediate HDL levels were significantly higher in ACS patients compared to those registered in healthy control subjects (51.67 ± 16.37% and 42.25 ± 13.95%, respectively, *p* < 0.0001). The same results were obtained for small HDL, i.e., a significant increase in small HDL in ACS patients compared to healthy control subjects (0.046 ± 0.04% vs. 0.075 ± 0.05%, respectively, *p* < 0.001) for intermediate HDL levels.

## 4. Discussion

Hypercholesterolemia is defined as an increase in LDL or non-cholesterol HDL levels. It is also one of the most important risk factors for CVD. However, in addition to the increase in LDL levels, hypercholesterolemia is also characterized by an alteration of the LDL subclass distribution, with sdLDL particles predominating (average diameter < 25 nm) [15]. Interestingly, the increase in sdLDL levels, which is considered the most atherogenic parameter [16], is not limited to hypercholesterolemia but can occur in the presence of different CVD risk factors. For example, an increase in sdLDL levels occurs with type 2 diabetes [17], obesity, hypertension [18], smoking [19], and after menopause [20]. These data support the hypothesis suggesting that sdLDL levels could be a strong predictor and valuable marker for the occurrence of cardiovascular events. In the present study, we determined the distribution of LDL and HDL subclasses and sdLDL levels in North African ACS patients and in healthy control subjects and compared the sdLDL levels to the established biomarker used for the estimation of the cardiovascular risk. Our results showed that the distribution of LDL in ACS patients was altered compared to healthy control subjects, with a significant increase in sdLDL. These results agree with other studies reported in the literature showing that sdLDL levels are higher in patients at high cardiovascular risk. In addition, studies have shown that an increase in sdLDL levels increases the risk of CHD in higher risk patients than in patients with lower or moderate risk of CVD but with lower sdLDL levels [21,22].

sdLDL measurements have even been suggested to be a valuable biomarker for estimating the future onset of CVD beyond the predefined cardiovascular risks [21,22]. Nevertheless, there is not yet a clear consensus regarding the threshold level of sdLDL that can be associated with a higher risk of cardiac events. Qi et al. have shown that an sdLDL level of 2.31 mmol/L is associated with a 70% increase in the rate of progression of atherosclerotic plaque [6,7,8]. Duran et al. have shown that the risk of occurrence of cardiac events increases significantly with an sdLDL level of 1.28 mmol/L [6], and Higashioka et al. have shown that the risk increases significantly with an sdLDL level as low as 0.90 mmol/L [21]. Other studies have reported an increase in the incidence of cardiovascular events for lower sdLDL levels (for instance, 0.60 mmol/L) [21,22,23,24,25,26]. Our study is the first to investigate the level of sdLDL in a North African population and our results also show that there was a significant increase in the sdLDL levels of ACS patients. However, while the sdLDL levels of the ACS patients were approximately 13 times that of the healthy control subjects (0.303 ± 0.478 mmol/L vs. 0.0225 ± 0.043, respectively), they remained 2-to-3 times lower than those determined in previous studies [21,22,23,24,25,26]. This discrepancy between different studies with respect to the level of sdLDL that could be associated with a higher cardiovascular risk can be attributed to factors that include ethnicity, the methodology used to quantify plasma sdLDL levels and even the definition of the sdLDL fraction itself [27,28]. In addition, LDL is classified into four fractions based on their densities when separated through ultracentrifugation [29] or based on their sizes when separated via electrophoretic mobility [9], and into seven fractions when separated using polyacrylamide gel electrophoresis [7]. The results regarding sdLDL levels are thus not necessarily comparable, and these techniques have not been standardized to better define or identify the sdLDL fractions regardless of the method used. In the present study, we used the Lipoprint^®^ assay, which is the only diagnostic method certified by the FDA for the quantification of both LDL and HDL subclasses.

The sdLDL levels were compared to other markers commonly used in the evaluation of cardiovascular risk, particularly AIP, AC, CR-I and CR-II. Our results show that the four markers were abnormal for the ACS patients, which is consistent with the health status of these patients and confirms the usefulness of these atherogenic indices for the evaluation of CVD risk [30,31,32]. Interestingly, our results show that sdLDL levels were positively and significantly correlated with the AC and CR-I indices. Positive correlations, while low but significant, were also observed between sdLDL levels and the AIP and CR-II indices. These positive and significant correlations between sdLDL levels and four different atherogenic indices support the idea that measuring sdLDL levels would be a useful predictor of cardiovascular events. Moreover, while conventional indices such as AIP, AC, CR-I, and CR-II offer a glimpse of the severity of dyslipidemia, sdLDL measurements may provide further information for CVD risk assessment. To verify the usefulness of measuring sdLDL concentrations for predicting the incidence risk of CVD, we used ROC curve to analyze the predictive power of sdLDL. An ROC analysis is undeniably the most used method for analyzing the effectiveness of a diagnostic test. Our results show that sdLDL levels, based on AUC, displayed high sensitivity and specificity for predicting CHD (AUC 0.8437 95% CI 0.7780 to 0.9166, *p* < 0.0001), with an optimal cutoff value for sdLDL of 0.038 mmol/L. The powerful discriminative potential of sdLDL levels was comparable to that obtained with AIP (AUC 0.8566), AC (AUC 0.8362), CR-I (0.8311), and CR-II (AUC 0.8437). We compared our results to those obtained in different studies with different populations or ethnic groups. Similar results were obtained with an Indian cohort (AUC 0.83) [33], whereas with a Chinese cohort, sdLDL showed a discriminative potential with an AUC of 0.641 [34]. This discrepancy in the discriminative potential of sdLDL between studies can be attributed to several factors, including the type of analysis used to quantify sdLDL, and, likely, the population studied.

The HDL subclass analysis also showed that there was a significant alteration in the distribution of the 10 HDL-forming fractions in the ACS patients compared to the healthy control subjects. More specifically, the ACS patients showed a significant increase in small and intermediate HDL particles, with a significant decrease in large HDL particles. These results are consistent with elevated sdLDL levels in ACS patients. Although HDL are anti-atherogenic lipoproteins, their beneficial effect is dependent on the levels of the particles forming these HDL. The anti-atherogenic effect of HDL is mainly due to their cholesterol efflux capacity (CEC), which is also a metric measure of their functionality [35]. The CEC of HDL is inversely associated with the level of carotid atherosclerosis and with the risk of incidence of CHD, independently of HDL cholesterol levels [35]. The in vivo regulation of cellular cholesterol, particularly by macrophages, is mediated by the transfer of cholesterol toward preβ-HDL or S-HDL particles. This transfer contributes to the transformation of S-HDL into more mature HDL (L-HDL) that is rich in cholesterol, which, in turn, transfers this cholesterol to the liver for elimination. Although in vitro studies have shown that S-HDL has a greater CEC than other HDL fractions, clinical studies have shown that there is a significant decrease in the CEC of HDL and that the anti-atherogenic activity of HDL is significantly and inversely correlated with S-HDL levels. An increase in plasma S-HDL levels has been proposed as a marker to identify patients with unstable pectoris angina [36].

## 5. Conclusions

In conclusion, the results of the present study show a significant alteration in the distribution of LDL and HDL particles in ACS patients. This alteration consisted of an increase in the most atherogenic LDL particles, namely sdLDL. The ROC analysis showed that the level of sdLDL had a powerful discriminatory potential and could be used as a biomarker for cardiovascular risk. The discriminatory power was in the same range as other known atherogenic indices. HDL distribution in ACS patients was also altered, with a decrease in L-HDL and an increase in I-HDL and S-HDL. This alteration could affect the main anti-atherogenic activity of HDL, specifically their CEC, and thus promote a greater accumulation of cholesterol at the arterial wall.

However, the present study has some limitations, mainly the small sample size. Another limitation is the difficulty of accurately comparing our results with those of other studies due to differences in the techniques used to separate and quantify the sdLDL fraction and the ethnic background. Further studies are needed to clarify the usefulness of the sdLDL level as a valuable marker for CVD.

## Figures and Tables

**Table 1:** Baseline characteristic of acute coronary syndrome patients and control healthy subjects.

	Healthy Subjects	ACS Patients	*p*
N	100	205	
Sex (M/W)	52/48	125/80	0.14
Age, y	55.07 ± 5.88	57.47 ± 9.59	<0.0006
BMI, Kg/m^2^	24.33 ± 2.34	27.25 ± 3.73	<0.0001
DBP, mmHg	71.00 ± 6.11	77.02 ± 11.04	<0.0241
SBP, mmHg	120.90 ± 9.54	132.83 ± 15.99	<0.0001
Hypertension, %	0%	36.58%	<0.054
Glycemia, %	5.16 ± 0.50	8.21 ± 3.85	<0.0001
Diabetes, %	0	42%	<0.0001
Total cholesterol, mmol/L	3.75 ± 0.85	4.67 ± 1.19	<0.0001
HDL, mmol/L	1.26 ± 0.23	0.95 ± 0.23	<0.0002
LDL, mmol/L	2.85 ± 0.51	3.77± 0.98	<0.0015
Triglycerides, mmol/L	1.18 ± 0.31	2.12 ± 0.95	<0.0001
Smokers, %	0%	35%	<0.0002
Statin intake	0%	5%	<0.03
Familial history, %	0%	26%	<0.002
Plasma Atherogenic Index (PAI)	−0.04 ± 0.16	0.32 ± 0.28	<0.0001
Atherogenic coefficient	2.09 ± 0.84	4.24 ± 1.94	<0.0001
Castelli’s risk index I	3.09 ± 0.84	5.24 ± 1.94	<0.0001
Castell’s risk index II	2.33 ± 0.54	4.06 ± 1.94	<0.0001

BMI: Body mass index, DBP: diastolic blood pressure. SBP: systolic blood pressure.

**Table 2:** The AUC values of plasma sdLDL and of various atherogenic indices.

CVD Indices	AUC	Std. Error	95% Confidence Interval	*p*
AIP	0.8566	0.02083	0.8158 to 0.8975	<0.0001
AC	0.8362	0.02197	0.7932 to 0.8793	<0.0001
CR-I	0.8311	0.02197	0.7932 to 0.8793	<0.0001
CR-II	0.8730	0.01973	0.8343 to 0.9117	<0.0001
sdLDL	0.8437	0.03537	0.7780 to 0.9166	<0.0001

AI: Atherogenic index of plasma; AC: Atherogenic coefficient; CR-I: Castelli’s risk I; CR-II: Castelli’s risk II.

**Table 3:** Cutoff values at the optimum Youden’s index for different atherogenic indices and for plasma sdLDL.

CVD Indices	Cutoff Value	Youden’s Index	Sensitivity (%)	95% CI	Specificity (%)	95% CI
PAI	0.135	0.67	77.59	65.34% to 86.41%	88.14	77.48% to 94.13%
AC	4.350	0.51	51.72	39.16% to 64.07%	100	93.98% to 100.0%
CR-I	3.890	0.51	68.97	56.20% to 79.38%	81	72.22% to 87.49%
CR-II	3.195	0.61	67.24	54.42% to 77.92%	93	86.25% to 96.57%
sdLDL	0.038	0.60	70.69	57.99% to 80.82%	75.86	63.47% to 85.04%

AI: Atherogenic index; AC: Atherogenic coefficient; CR-I: Castelli’s risk I; CR-II: Castelli’s risk II.

## Data Availability

Not applicable.

## Supplementary Materials

The following supporting information can be downloaded at: https://www.mdpi.com/article/10.3390/biomedicines11041198/s1, Figure S1: Spearman correlation analysis between the sdLDL levels and several atherogenic indices.
