# Supplementary material for: Small Dense LDL Level and LDL/HDL Distribution in Acute Coronary Syndrome Patients
Source: Biomedicines. 2023 Apr 18;11(4):1198. doi: 10.3390/biomedicines11041198 (PMC10135780; doi:10.3390/biomedicines11041198)
Supplement: Supplementary file 1 [file biomedicines-11-01198-s001.zip › biomedicines-2313238-supplementary.pdf]

nutrients

MDPI

Small dense LDL as a valuable marker for cardiovascular disease

Alyann Otrante[1], Abdelghani Bounafaa[1,2], Hicham Berrougui[1,,3], Abdel-Khalid Essamadi[2], Michel Nguyen[4], Tamàs Fülöp[1] and Abdelouhed Khalil[1,*]

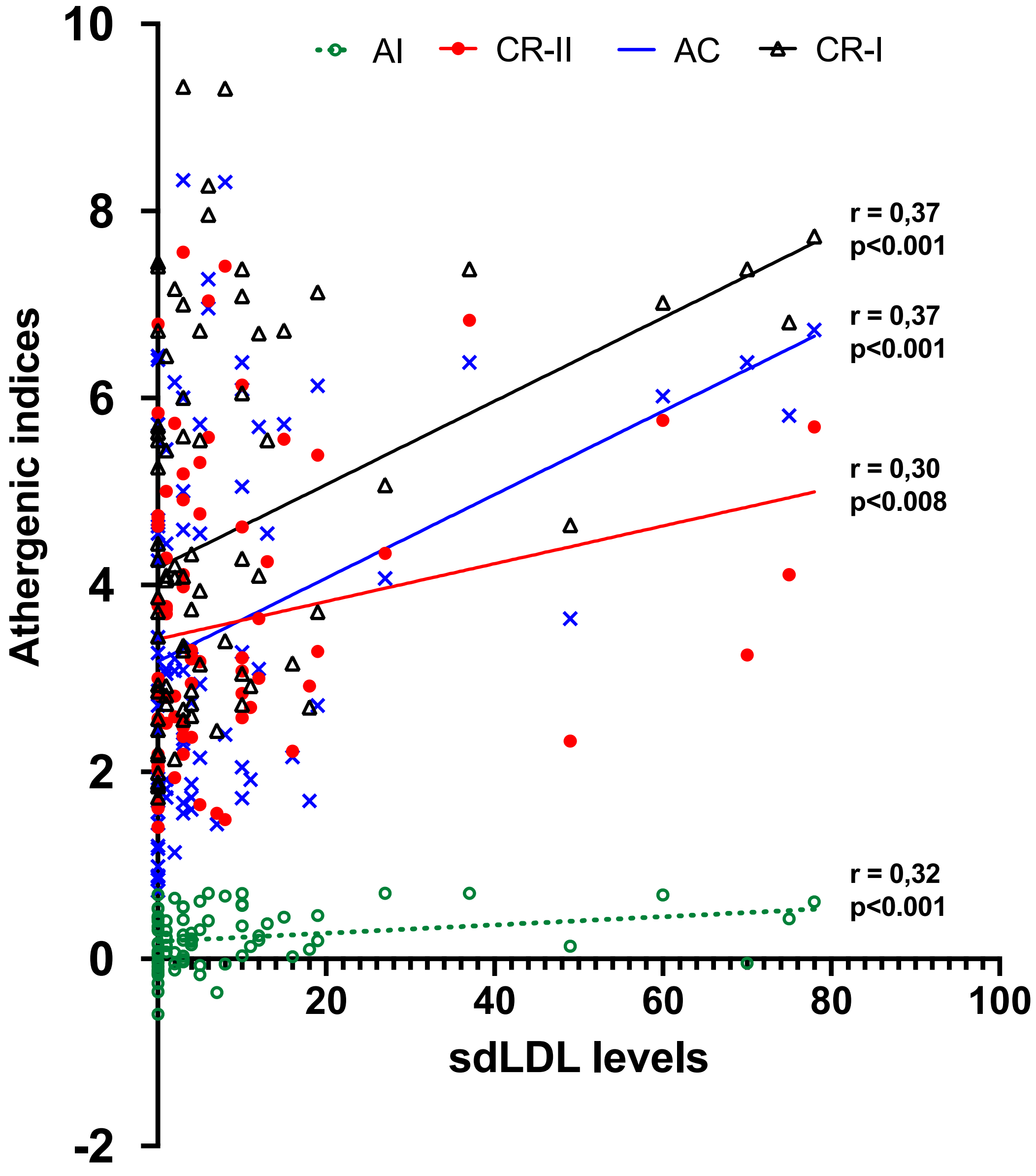

Figure S1: **Spearman correlation analysis between the sdLDL levels and several atherogenic indices.** AIP: atherogenic index of plasma, AC: atherogenic coefficient, CRI-I: Castelli's Risk Index and CR-II: Castelli's Risk Index
